# Novel electronic refreshers for cardiopulmonary resuscitation: a randomized controlled trial

**DOI:** 10.1186/1471-227X-12-18

**Published:** 2012-11-21

**Authors:** Stephen Magura, Michael G Miller, Timothy Michael, Robert Bensley, Jason T Burkhardt, Anne Cullen Puente, Carolyn Sullins

**Affiliations:** 1 The Evaluation Center, Western Michigan University, 1903 W. Michigan Ave., Kalamazoo, MI, 49008, USA; 2 Department of Human Performance and Health Education, Western Michigan University, 1903 W. Michigan Ave., Kalamazoo, MI, 49008, USA; 3 During the study: The Evaluation Center, Western Michigan University, 1903 W. Michigan Ave., Kalamazoo, MI, 49008, USA

**Keywords:** Cardiopulmonary resuscitation, CPR, CPR refreshers, Prehospital emergency care, Cardiac arrest

## Abstract

**Background:**

Currently the American Red Cross requires that individuals renew their cardiopulmonary resuscitation (CPR) certification annually; this often requires a 4- to 8-hour refresher course. Those trained in CPR often show a decrease in essential knowledge and skills within just a few months after training. New electronic means of communication have expanded the possibilities for delivering CPR refreshers to members of the general public who receive CPR training. The study’s purpose was to determine the efficacy of three novel CPR refreshers - online website, e-mail and text messaging – for improving three outcomes of CPR training - skill retention, confidence for using CPR and intention to use CPR. These three refreshers may be considered “novel” in that they are not typically used to refresh CPR knowledge and skills.

**Methods:**

The study conducted two randomized clinical trials of the novel CPR refreshers. A mailed brochure was a traditional, passive refresher format and served as the control condition. In Trial 1, the refreshers were delivered in a single episode at 6 months after initial CPR training. In Trial 2, the refreshers were delivered twice, at 6 and 9 months after initial CPR training, to test the effect of a repeated delivery. Outcomes for the three novel refreshers vs. the mailed brochure were determined at 12 months after initial CPR training.

**Results:**

Assignment to any of three novel refreshers did not improve outcomes of CPR training one year later in comparison with receiving a mailed brochure. Comparing outcomes for subjects who actually reviewed some of the novel refreshers vs. those who did not indicated a significant positive effect for one outcome, confidence for performing CPR. The website refresher was associated with increased behavioral intent to perform CPR. Stated satisfaction with the refreshers was relatively high. The number of episodes of refreshers (one vs. two) did not have a significant effect on any outcomes.

**Conclusions:**

There was no consistent evidence for the superiority of novel refreshers as compared with a traditional mailed brochure, but the low degree of actual exposure to the materials does not allow a definitive conclusion. An online web-based approach seems to have the most promise for future research on electronic CPR refreshers.

## Background

Widespread effective training in cardiopulmonary resuscitation (CPR) can save countless lives. Nearly 80% of cardiac arrests are witnessed by a family member and occur in one’s home. The survival rate of victims of sudden cardiac arrest may be no more than five percent, because the overwhelming majority of bystanders who witness the event do not know how to perform CPR [1]. Less than one-third of victims of sudden cardiac arrest receive CPR from bystanders, and even fewer receive adequate quality CPR [2]. Often lay responders, despite having a desire to provide basic life support, lack the skill to correctly provide this service [3]. Moreover, training alone may not be enough to ensure that individuals are willing and able to effectively administer CPR [4-6].

Those who have been trained in CPR may show a decrease in essential knowledge and skills within just a few months after training [7-12]. Further, lack of confidence in conducting CPR [13], as well as lack of willingness to attempt it [14], may be impediments to intervening in a crisis. Currently the American Red Cross requires individuals to renew their CPR certification annually; this often requires a 4- to 8-hour refresher course. However, it is neither feasible nor cost-effective to conduct frequent in-person recertification courses.

Studies show that frequent refresher courses can help both medical and lay personnel maintain CPR skills [15-19]. Smaller-scale refresher materials, presented to trainees between certifications and re-certifications, could fill in memory and confidence gaps, but only if the effort is made to use them [20,21]. Poster-based refreshers were equally as effective as instructor-led refreshers in relation to skill retention one year post training [22]. Moreover, additional training for people previously certified in CPR led to lesser declines over time in willingness to perform CPR [23].

Actual skill is only one of the factors that can make the difference between a passive bystander during an emergency and an effective administrator of CPR. Social-cognitive theory emphasizes that enacting any behavior also depends on a person’s confidence (“self-efficacy”) in performing that behavior and motivation (intent) to perform it [24,25]. Program evaluators have recognized the gap between the acquisition of knowledge or skills and subsequent changes in attitudes and behavior [26]. In support of the social-cognitive theory of behavior change, a recent study of motivation, self-confidence and skill retention found that gains in these factors were dependent on the method and timing of CPR training [27]. Therefore, an effective CPR refresher must address not only skill retention, but also confidence and behavioral intention to perform CPR. More research is required to examine the effectiveness of CPR refreshers on skill retention, confidence and motivation, as well as the appropriate format, timing and frequency of such refreshers.

In order to be effective, CPR refreshers should be easily accessible, available at no or low cost, and likely to be reviewed by trainees in the general population. Thus, the most desirable format would be to deliver the content to a trainee’s home or office, rather than requiring the person to attend a session in a special location. New electronic means of communication have expanded the possibilities for delivering CPR refreshers to members of the general public who received training in CPR. Such an approach does not include renewed CPR practice, which is difficult to arrange. Our basic assumption was that various electronic modalities can actively direct the attention of prior trainees to messages designed to help them in recalling correct CPR techniques. Specifically, electronic refreshers are hypothesized to aid in retaining CPR administration skills, confidence in performing CPR and intention to perform CPR when needed.

General population access to and use of electronic communication is already quite extensive, especially among younger people, and is continually increasing [28-32]. Studies have documented internet access among even more difficult to reach (e.g., low income) populations [33-35]. Based on these trends of increasing access to electronic and mobile communications, the novel CPR refresher formats selected for this study were: online website, e-mail, and text messaging by cell phone. Recent studies have shown that such electronic communication formats can be effective in increasing confidence and motivation to engage in health promoting behaviors [36-39].

The present study conducted a randomized controlled trial (RCT) of four CPR refreshers - online website, e-mail, text messaging and a mailed brochure – to determine their efficacy in affecting skill retention, confidence in using CPR and intention to use CPR at a one year follow-up after initial CPR training. The first three refreshers, based on electronic communication, can be considered “novel” in that they are not typically used to refresh CPR knowledge and skills. Print materials, which are often costly to produce, have been the traditional method for delivering many health behavior messages to the home. The mailed brochure is a common health education format that was conceptualized as the control condition for the study.

Correctly recalled and applied CPR steps are essential in lifesaving. Studies have shown that the quantity and spacing of refreshers is related to the degree of subsequent skill retention [40,41]. Thus the present study also tested whether repeating the CPR refresher episodes was useful for proper sequencing of skills in administering CPR.

The primary study hypothesis is that the three novel, technologically “active” CPR refreshers, will yield outcomes superior to those of the relatively passive, traditional mailed brochure refresher. (We do not hypothesize the relative efficacy of the three novel refreshers; this aspect of the analysis is exploratory.) The secondary study hypothesis is that subjects receiving two refresher episodes will have outcomes superior to subjects receiving only a single refresher episode.

## Methods

### Interventions

Using clear, concise visual depictions, Each of the four refreshers described below reviewed the five principal CPR skills: (1) checking for responsiveness, (2) calling 911, (3) ventilation, (4) compression, and (5) hand placement. Each refresher included separate visual and narrative (written and/or audio) depictions of procedures for conducting CPR. To avoid information overload for the subjects, we limited the content of the refreshers and subsequent re-tests to adult CPR procedures.

#### Online website

Subjects received an e-mail with a link to the CPR refresher website, which consisted of 10 modules. Users were presented with refresher information and scenarios based on their unique learning profile driven by the above theoretical constructs. The application of these skills to a situation of bystander assistance was followed by a review of the five basic skills. Additional guided experiences were presented, allowing the subject to apply their knowledge, skills, readiness, efficacy, and intent to various scenarios. The approach used within this intervention differs significantly from that which would be found in a traditional educational course approach. It was not the intent of the website to replicate or serve as an educational course, as online CPR courses currently exist. Rather, the basis of the website is dynamic in the sense that users are directed toward refresher information that is keyed to their stated degree of confidence in being able to perform actions associated with the five CPR skills. The website focused on review of skills based on where the user was in relation to their confidence to engage in CPR administration. Prior to this, no web-based approach had been developed that provides refresher intervention which is stage-based and designed to enhance intent to engage in CPR administration behaviors.

#### E-mail

A series of 12 e-mail messages was sent to subjects over the span of one month. E-mail messages were short in nature and focused on the five basic CPR skills, confidence to perform CPR, and intent to intervene in CPR situations. It was determined that e-mail would serve as a better refresher modality than automated voicemail on cell phones due to the ability to deliver more appropriate content, reduced constraints due to length of message, and ability to archive for further review, which is consistent with the other two novel refresher formats.

#### Phone text messages

A series of three text messages over the span of three days was delivered to SMS-enabled cell phones. The messages were short in nature and focused on the five basic CPR skills, confidence and readiness to intervene in CPR situations. Messages were delivered during the same time period as other refresher formats.

#### Brochure

A full-color brochure was mailed to subjects. The print material design consisted of a multi-panel brochure and was based on the same content as used for the electronic interventions. However, the print material refresher was static in nature, rather than interactive as can be achieved in electronic learning approaches. The brochure used similar images and narratives as the novel refreshers. The mailed brochure was the most passive of the refreshers and thus was considered the control condition for this study.

### Study sites

Three sites served as the subject pool for this study. These sites were selected on the basis of their closed environments; relatively stable populations; likelihood of obtaining participants; and diversity in gender, race, ethnicity, and geographic locations. The sites were also diverse in the type of learner that was sampled – site 1 was primarily academic (students enrolled at Western Michigan University), site 2 was primarily professional (primarily individuals who worked in various professions within the greater Kalamazoo, MI county area), and site 3 contained a mix of professional and academic learners, either Utah University students and staff or working professionals within the surrounding Salt Lake City community. At each site, an instructor who underwent training with the research team solicited participants, mainly by posted fliers or company-based recruitment meetings.

### Study procedures

#### Initial CPR training

All participants received the same initial CPR training (a 4-hour course) following the guidelines of the American Red Cross at each site. The training followed the standard format for lay rescuers and included adult, child and infant CPR and automated external defibrillator (AED) training. However, the refreshers and retest focused on adult sudden cardiac arrest, as that is the most likely scenario participants will face. We also excluded AED refresher training. All instructors were recognized instructors through either the American Red Cross or American Heart Association. The initial CPR training was administered in small group format (about 12 participants each). Participants received Adult CPR certification cards upon successful completion.

#### Trial 1: effect of refresher format

Participants received initial CPR training and an immediate post-test (questionnaire and CPR skills assessment; see below). Participants were randomly assigned to one of the four refreshers based on a previously generated random assignment schedule. Any individuals who did not have access to the technology to participate in their assigned refresher condition were dropped from the study. Attempts were made to provide all subjects with their assigned refreshers six months after completing the initial CPR training. Individuals who could not be contacted by e-mail (due to incorrect or obsolete e-mail address) or whose text message service rejected the text messages were dropped from the study analysis. Participants were contacted and invited for a re-test (questionnaire and skills assessment) at the end of month 12 after initial CPR training. Trial 1 was conducted during September 2009 – December 2010.

#### Trial 2: effects of refresher format and frequency

In Trial 2, begun after the Trial 1 CPR training was completed but before any analysis was conducted, a new sample of subjects received initial CPR training and the post-test. The same sets of refreshers were offered twice, at 6 and 9 months after initial CPR training, instead of once as in Trial 1. Nine month refreshers were sent out irrespective of whether the subjects reviewed the six month refreshers. Participants were contacted and invited for a re-test at 12 months after initial CPR training. Trial 2 was conducted during January 2010 – April 2011.

#### Human subjects protection

The study was approved by the Human Subjects Institutional Review Board of Western Michigan University.

### Sample

The study’s goal was to accumulate complete data on at least 60 subjects per intervention condition over the two independent trials, for a total sample of 480 participants. The number of individuals who were recruited and received CPR training was 680. Of those 680 individuals, 23 were dropped from the study due to returned e-mails, rejected text messages, or no e-mail capability; 11 had data entry errors on important variables that were not correctable; and 16 cases were dropped due to receiving an erroneous third refresher intervention; this left 630 cases. Of these 630 cases that were eligible for 12 month post-refresher follow-up, 304 individuals failed to complete the follow up testing or did not provide sufficient information on the follow-up to compute scores on the three outcome variables. This left 326 individuals available for data analysis, which was 51.7% (326/630) of the sample eligible for follow-up (See Table 1).

**Table 1:** Number of Participants by Refresher Type by Trial in the Analysis (n=326)

		**Type**							
			**Brochure**	**E-mail**		**Text Message**		**Website**	
		**N**	**% of assigned**^**1**^	**N**	**% of assigned**	**N**	**% of assigned**	**N**	**% of assigned**
Trial	1	52	(63%)	57	(66%)	31	(42%)	48	(55%)
Trial	2	21	(23%)	50	(55%)	30	(34%)	37	(41%)

^1^Percent of the total number of subjects who were randomly assigned to each refresher type within each trial.

### Measures

#### Post-test and re-test

Immediately after CPR training was completed, the one-on-one CPR skills test was performed (post-test). Infant CPR, child CPR, and/or AED training was included only if these were requirements for certification at a particular site. Each participant took the skills test in a partitioned area to eliminate any bias that may occur as a result of watching a fellow participant. Surveys measuring confidence in CPR and intent to conduct CPR were administered after each skills test as part of the post-test procedure. At one year after initial CPR training and receipt of refreshers, subjects were contacted by their instructors to return to the original training site for the re-test, which had the same content as the post-test. Participants received $15 for completing the re-test.

#### Adult CPR skill sheet (observation of performance)

CPR skills were assessed by the instructor based upon a skill sheet for adult CPR as specified by the American Red Cross. This consisted of 39 observational items measuring CPR-related skills across two cycles of CPR performance. The total correct out of 39 possible was the score used in the statistical analyses. This method of testing, when scored by persons with expertise in CPR, has been shown to be a reliable method of measuring CPR skills [42].

#### CPR confidence assessment

This scale was computed from nine fixed response items answered by the participants, e.g., “how confident would you be about performing CPR if the victim still showed signs of life?” Each item was rated using the following responses: “not at all confident” (=0), “slightly confident” (=1), “moderately confident” (=2), mostly confident (=3), and “totally confident” (=4). The respondent’s scores were averaged across the nine items to produce a continuous confidence score ranging from “0” (lowest confidence) to “4” (highest confidence), with internal consistency reliability (alpha) = 0.93.

#### Behavioral intent to perform CPR

The behavioral intent scale was based on a reduced set of 10 items from an original set of 21 Likert-type items, e.g., “how would you feel about responding to an emergency if the victim was a complete stranger?” Each item was rated using the following responses: “definitely not” (=0), “probably not” (=1), “not sure” (=2), “probably yes” (=3), and “definitely yes” (=4). Psychometric examination reduced these 21 items to 10 items, whose responses were averaged to produce a continuous behavioral intent score ranging from “0” (lowest behavioral intent) to “4” (highest behavioral intent), with internal consistency reliability (alpha) =0.89.

#### Satisfaction with the refreshers

This consisted of 9 Likert-type survey questions completed at re-test, e.g., “the refresher I received was helpful for refreshing my CPR skills; this CPR refresher made me feel more confident about acting in the case of an emergency”.

#### Exposure to the refreshers

Measures of actual exposure to the refreshers were created by coding “1” for individuals who had at least one indication of interaction with the refresher (at least one e-mail opened, one text message response, or one website visit). These were determined by electronic tracking of subject behavior. It was not feasible to use a more detailed measure of exposure because of the difficulty of creating commensurate measures for the different refreshers, as well as the relatively low percentage of each group who actually viewed any of the refresher material. Since we did not have an adequate indicator whether the mailed brochure was reviewed, there is no separate exposure variable for the brochure. Although subjects were asked on the re-test interview whether they reviewed the brochure, there seemed to be some confusion between the brochure and the CPR “card” (actually a small tri-fold pamphlet) that subjects received at the end of the initial training; some subjects seemed to have reviewed the latter, but identified it as the “brochure”. In any event, all the brochures mailed appeared to have been delivered; there are no reports of any being returned by the post office (they were mailed first class).

These exposure variables were then used to create a coding system that resulted in three indicator- coded groups for the regression analyses: a brochure-only group; a group that was assigned to a novel refresher, but did not show exposure (no opened e-mails, no text message responses, etc.); and a group that was assigned to a novel refresher and showed exposure (opened at least one e-mail, responded to at least one text message, etc.). The reference category to examine effects in this analysis is “received brochure”. The subsample sizes for each refresher condition for the exposure analysis are in Table 2, which also indicates the percent of those assigned to each novel refresher who were exposed to that refresher.

**Table 2:** Indicator Variables for Refresher Exposure Analysis (both trials, n=276)

** Refresher**	**Received Brochure**	**Assigned to Novel Refresher– No Exposure**	**Assigned to Novel Refresher – Exposure**	**Percent Exposed**^**1**^
Brochure	73	0	0	-
E-mail^2^	0	16	41	72%
Text Message	0	27	39	59%
Website	0	51	29	36%

^1^(Number exposed divided by total number assigned to that novel refresher) X 100.

^2^Trial 2 e-mail group not included (see text).

### Statistical analysis

#### Refresher intent to treat (ITT) analysis

The purpose of the ITT analysis was to measure the impact of refresher type and frequency on the skill level, confidence and behavioral intent of the subjects at the one year re-test. In this analysis, all individuals assigned to a refresher are included; this achieves an unbiased estimate of intervention effect [43]. The subsample sizes for each refresher condition for the ITT analysis are shown in Table 1. A respondent’s age, education, ethnic category, gender, trial (1 or 2), trial by refresher interaction, and post-test score were entered as covariates in multiple regression analyses, conducted separately for each of the three outcomes.

#### Refresher exposure analysis

Since not all subjects were actually exposed to the refreshers (i.e., saw or reviewed them), a second type of analysis was conducted to examine the impact of *actual exposure* to a particular refresher on the three outcomes, as compared with the brochure group. The exposure data for the Trial 2 e-mail group was missing due to an error in the e-mail tracking process; we could not verify that these e-mails were opened. However, there was evidence that the Trial 2 e-mail group did in fact receive the e-mail refreshers; thus this group was included in the intent to treat analysis only.

A respondent’s age, education, ethnic category, gender, trial (1 or 2), trial by refresher interaction, and post-test score were entered as covariates in multiple regression analyses for exposure, conducted separately for each of the three outcomes. Note that, in contrast to ITT analysis, exposure analysis has the disadvantage of being vulnerable to subject selection effects which may bias the results, despite statistical controls for potential confounding variables [43].

## Results

### Descriptive statistics

The socio-demographic characteristics of the sample are given in Table 3; these reflect the characteristics of the study site populations. Table 3 also shows the post-test and retest scores on the three outcome measures. Skills, confidence and behavioral intent to perform CPR all declined for the sample as a whole between the post-test and re-test.

**Table 3:** Sample Descriptive Statistics (N=326)

	
Age (mean years ± SD)	35.1 ± 14.9
Ethnicity (%)	
White	87.3
Black	4.6
Hispanic	2.1
Other	6.0
Gender	
Female	50.6
Male	49.4
Education (%)	
Graduate degree	15.6
Four year college graduate	21.5
Two year college graduate	14.4
High School graduate/GED	45.4
Less than high school	1.8
Missing data	1.3
CPR Skills (mean ± SD)	
Post-test	35.75 ± 3.97
Re-test*	30.33 ± 6.51
Confidence to Perform CPR (mean ± SD)	
Post-test	3.39 ± 0.53
Re-test*	3.13 ± 0.54
Behavioral Intent to Perform CPR (mean ± SD)	
Post-test	2.89 ± 0.66
Re-test*	2.42 ± 0.61

### Intent to treat (ITT) analysis

To test the primary hypothesis, the subjects assigned to any of the three novel refreshers in trials 1 and 2 were combined into one group and compared with the subjects who were assigned to the brochure. If an overall effect were to be found, post-hoc tests would be conducted to localize the source of the effect.

#### ITT CPR skills

The marginal means and standard errors (in parentheses) for skill score at the 1 year re-test for the brochure group were 23.9 (3.73) and for the pooled novel refresher group were 23.9 (0.71), which was not significant (p > .05). This indicates that there was no effect on skill retention for the novel refreshers as a group as compared with the brochure. Significant predictors (p < .05) in this model were age (the younger, the more skill retention), education (the more education, the more skill retention), and being White (more skill retention than other ethnic categories), post-test score (the higher the skills at post-test, the higher at re-test). In all, 18.9% of the total variance in skills at re-test was explained by this model.

#### ITT confidence to perform CPR

The marginal means and standard errors for the confidence score at the 1 year re-test for the brochure group were 1.86 (0.22) and for the pooled novel refresher group were 1.89 (0.06), which was not significant (p > .05). This indicates that there was no effect on confidence score at re-test for the novel refreshers as a group as compared with the brochure. Significant predictors (p < .05) in this model were age (the younger, the higher the confidence); education (the more education, the higher the confidence), gender (females had higher confidence), and post-test score (the higher the post-test, the higher the re-test). In all, 28% of the total variance in confidence at re-test was explained by this model.

#### ITT behavioral intent

The marginal means and standard errors for behavioral intent at the 1 year re-test for the brochure group were 1.00 (0.17) and for the pooled novel refresher group were 1.10 (0.07), which was not significant (p > .05). This indicates that there was no effect on behavioral intent at retest for the novel refreshers taken as a whole. The only significant predictor (p < .05) in this model was the post-test score (the higher the post-test, the higher the re-test); 34.7% of the total variance in behavioral intent score at re-test can be explained by this model.

The effect of one vs. two episodes of refreshers (“trial”) was also not significant for any of the outcomes, nor was the interaction of refresher type by trial. In addition, we conducted exploratory analyses to identify possible effects of individual types of refreshers on outcomes, individually for trials 1 and 2. No significant effects for any of the three novel refreshers vs. the brochure were found.

### Exposure to refreshers analysis

In these analyses we constructed six regressions, two for each outcome variable. The first regression for each outcome made two comparisons (“contrasts”) using indicator variables: novel refresher exposure vs. brochure and no novel refresher exposure vs. brochure (brochure was the reference category). The second regression for each outcome made the comparison of novel refresher exposure vs. no novel refresher exposure (the latter was the reference category). Of the nine refresher effect comparisons, only one was statistically significant: confidence was higher for novel refresher exposure vs. no novel refresher exposure for confidence to perform CPR at re-test (p < .01).

Lastly, we conducted exploratory analyses to identify possible effects of exposure to individual refresher formats on outcomes. Only one significant effect was found; the mean on behavioral intent for the group of respondents exposed to the website was significantly higher than for the brochure group (p < .01).

### Satisfaction with refreshers

Participants were asked about their satisfaction with various aspects of the CPR refresher process at the 1 year follow-up. Table 4 gives the results of the answers to the individual questions, classified by refresher type. A one-way analysis of variance was conducted to examine whether responses to the questions differed significantly by refresher type; this analysis is exploratory. For items 1 – 6, the responses were coded from strongly disagree = 1 to strongly agree = 5. For items 7–9, the responses were coded no = 1 and yes = 2. The figures in the table are the mean scores for each refresher category. There was a significant difference in the responses for the refreshers for items 1–6, but not 7–9.

**Table 4:** Satisfaction with CPR Refreshers by Type of Refresher Assigned

		**Group Means**			
**Question**	**ANOVA omnibus test (f)**	**Brochure**	**E-mail**	**Text message**	**Website**
1. The CPR refresher was helpful for refreshing my CPR skills.	11.90***	3.39	3.91	2.90	3.00
2. This refresher made it easy to obtain CPR related information.	7.47**	3.34	3.96	2.94	3.11
3. The CPR refresher will help me if I ever happen to be faced with a CPR emergency.	11.298***	3.42	3.89	2.92	3.00
4. The CPR refresher made me feel more confident about acting in the event of an emergency.	7.850**	3.33	4.13	2.95	3.02
5. This CPR refresher helped me remember how to perform at least one CPR skill that I had forgotten how to perform.	8.03**	3.28	3.98	2.92	2.92
6. I would recommend the CPR refresher I received to other individuals wanting to refresh their skills.	9.76**	3.48	4.36	2.87	3.12
7. Are there any other issues that may prevent you from responding to an emergency that were not addressed in the CPR refresher you received?	0.064	1.06	1.04	1.04	1.08
8. Is there anything that you would have changed about the CPR refresher you received?	.202	1.22	1.30	1.33	1.17
9. Is there anything else you would like to share about your experience with the CPR refresher you received?	.471	1.12	1.20	1.18	1.08

*** p ≤ .001, ** p ≤ .01, * α≤ .05.

### Use of CPR during study period

Five subjects said they performed CPR during the one year period following CPR training, four of whom stated they had done so after having received refreshers. Comments made were: the refresher helped because “it was fresh in my mind”; the refresher made him/her feel capable when performing CPR; and “the refreshers helped me”.

## Discussion

There was no evidence that assignment to any of three novel refreshers improved outcomes of CPR training one year later in comparison with receiving a mailed brochure refresher. This held for all three outcomes examined: CPR skill retention, confidence for CPR, or intent to help in a cardiac emergency. However, interpreting this “intent to treat” result is difficult because many subjects did not actually review the electronic refreshers that were sent. Comparing outcomes for those exposed to the electronic refreshers vs. those not exposed indicated a significant effect for one of the three outcomes, confidence in performing CPR. According to social-cognitive theory, because increased confidence in being able to perform a behavior should increase the likelihood of performing that behavior, there is at least a potential that the novel refreshers can influence whether the subjects would conduct CPR in an emergency.

The study identified a significant effect of refresher website exposure specifically on increased behavioral intent. The website refresher can be considered more interactive than the other novel refreshers. The algorithm-based web program engaged the subject in critical thinking, leading them to appropriate responses in contrast to the other refreshers which were more didactic in their approach to reviewing CPR technique. The greater degree of active engagement in reviewing the principal CPR skills made possible by the website format may be responsible for the more positive outcome of this refresher compared with the others. This result bears more investigation, although of course it may be a chance finding, given the multiple comparisons made in the exploratory analyses.

The number of refresher episodes (one vs. two) did not show a significant effect on any outcomes. This indicates that repeating the refreshers during a one year post-training period is not an effective strategy for retaining CPR capability.

Examining the pattern of the satisfaction data, highest satisfaction occurred for the e-mails, second highest for the brochure, third highest for the website, and lowest for the text messaging. Those who received e-mails also had the highest rate of exposure to any of the novel refreshers, measured by whether they opened any refresher e-mails. From these data, we might conclude that e-mail was the most successful of the novel CPR refreshers, at least in terms of subject acceptance of and reactions to the refresher. It is possible, however, that a higher proportion looked at the mailed brochure than viewed any of the novel refreshers, although the data are ambiguous on this point, because of possible confusion with the CPR reminder “card” received at the initial training.

One predictor model determined that age (younger), education (higher) and race (White) were significant predictors of skill retention, although these variables only accounted for 19% of the variation in skill. A possible explanation is that younger, more educated and White trainees have more recent or extensive exposure to educational settings that prime or prepare them to better assimilate additional learning such as CPR training.

One drawback of the novel refreshers is that they may be treated as SPAM by recipients, given the large numbers of electronic contacts that individuals typically receive. Also, there is a natural tendency to ignore information that is not immediately salient, as when there is no immediate need to perform CPR. In that respect the modest, “low tech” CPR reminder card may be a superior device – the subject carries it in their purse or wallet and can refer to it when and if the occasion for performing CPR arises. The four refreshers utilized in this study do not enable immediate access to information at the precise time it’s needed.

### Study limitations

The relatively high attrition rate of subjects for follow-up testing may have contributed to a reduction in the power of the statistical tests for refresher effects. Many of the subjects, particularly the students, were difficult to re-contact, and the study’s resources were not sufficient for intensive, repeated follow-up efforts.

The study was limited in being able to document the degree of actual exposure to the refreshers. Originally, data were collected on the number of e-mails and pdf files opened by the e-mail group, the number of text messages responded to by the text message group, and the number of website “nodes” visited by the website group. However, the recording of the “node information” made detailed exposure analysis unfeasible, and as such, the exposure variables for all novel refresher groups were restricted to a dichotomous indicator (no exposure vs. some exposure). On balance, inspection of the partial data on refresher exposure available indicates that most subjects did not review most of the electronic refresher material sent. Thus, it is possible that the novel refresher approach could be more effective if subjects could be encouraged to review more of the material when it is sent to them.

### Directions for future research

Additional research on novel electronic refreshers seems justifiable, given our finding that novel refreshers may affect prior trainees’ confidence in performing CPR and that exposure to an online website refresher appeared to affect intent to perform CPR. Further research with a website refresher may be most promising, because this allows for greatest interactivity with the subject and would provide excellent access due to internet availability on smartphones, a technology that was still rare when the current study was designed.

The finding that age, educational level and ethnicity were related to retention of CPR skills could lead to further research investigating the reason(s) for these relationships.

Also, it might be fruitful to conduct a study of individuals who have found themselves in situations where CPR needed to be done, to see what they did or didn’t do, and determine what kinds of refreshers, technology, motor skills training or other support helped them to perform CPR correctly.

## Abbreviation

CPR: Cardiopulmonary resuscitation.

## Competing interests

The authors declare they have no competing interests.

## Authors’ contributions

MGM, TM and RB developed the interventions and research instrumentation, planned and managed the data acquisition, and contributed to the intellectual content and revision of the manuscript. SM was a co-investigator who contributed to the intellectual content of the study, planned the statistical data analysis and wrote the manuscript. CS contributed substantially to the research design and portions of the manuscript. ACP and JTB conducted the statistical data analysis at different periods of the study and contributed to its interpretation. All authors have read and approved the final manuscript.

## Pre-publication history

The pre-publication history for this paper can be accessed here:

http://www.biomedcentral.com/1471-227X/12/18/prepub
